# Proteomic characterization of clinical *Candida glabrata* isolates with varying degrees of virulence and resistance to fluconazole

**DOI:** 10.1371/journal.pone.0320484

**Published:** 2025-03-25

**Authors:** Pamela El Khoury, Ahmad Zeidan, Roy A. Khalaf

**Affiliations:** Department of Biological Sciences, Lebanese American University, PO Box 36, Byblos, Lebanon; Cleveland Clinic Lerner Research Institute, UNITED STATES OF AMERICA

## Abstract

*Candida glabrata*, an opportunistic fungal pathogen, is a significant contributor to mortality among individuals with weakened immune systems. Antifungal drugs such as azoles work by inhibiting the Erg11 enzyme, altering the conversion of lanosterol to ergosterol. Resistance to azoles is increasing among *Candida* species worldwide, and in Lebanon. This study aims to determine the identity of cell wall proteins that could be involved in resistance and virulence in *Candida glabrata* Lebanese hospital isolates. Four isolates with varying degrees of resistance and virulence to fluconazole were subjected to proteomic analysis. Cell wall proteins of each isolate were extracted and analyzed using MALDI TOF TOF mass spectrometry to identify proteins responsible for virulence and resistance under exposure to fluconazole. Results showed the exclusive presence of efflux pumps such as Cdr1 and Pdr1 after exposure to fluconazole, in addition to other resistance mechanisms such as activation of multidrug transporter proteins and specific response pathways such as the RIM 101 pathway that could be involved in drug resistance and adhesion. Proteomic profiling exhibited proteins differentially detected in the virulent isolates such as the autophagy related proteins Atg 11 and Atg16, and stress response proteins Sgf11 and Alg2. In conclusion, our study suggests several mechanisms that contribute to resistance and virulence in *C. glabrata*.

## Introduction

*Candida glabrata (C. glabrata)*, an asexual haploid yeast species, is an opportunistic human fungal pathogen [1]. It is the second or the third most common cause of candidiasis depending on the site of infection [2]. *C. glabrata* has a genome size of about 12.3 million base pairs, divided across 13 chromosomes [3]. It contains around 5,293 genes, but only some have been studied and confirmed to encode proteins [4]. Furthermore *C. glabrata* cells are only 1-4 µm in diameter, remarkably smaller than other *Candida* species [5]. *Candida* species are known to be polymorphic, i.e., able to transition between distinct morphological states [6]. Yet, *C. glabrata* has never been observed in a filamentous form, making it easily distinguishable from other *Candida* species observed in medical practice [7]. However, under nitrogen limiting conditions and carbon dioxide exposure it can develop pseudo hyphal structures [8,9]. Thus, despite being a non-dimorphic yeast, *C. glabrata* is still capable of being highly virulent [10].

The fungal cell wall is a vital dynamic structure for growth, survival, morphogenesis, pathogenicity, and defense against osmotic and mechanical stressors since it is the first entity interacting with the host with adhesive properties and complex structure consisting of polysaccharides, proteins, and lipids [11]. Polysaccharides and proteins are covalently cross-linked to each other and are organized into layers: chitin and β-1,3-glucan that make the homogenous inner layers, and an external layer of beta 1-6-glucans to which most glycoproteins, including the adhesins and proteases, are attached [12]. Many of the cell wall proteins (CWPs) in *C. glabrata* are glycosylphosphatidylinositol (GPI)-anchored proteins that allow it to interact with the host and environmental conditions [13].

One of the key features of many pathogenic fungi is their ability to adhere to host surfaces, and *C. glabrata* excels in adhesion by encoding large sets of surface-exposed CWPs in its genome, most importantly the adhesin Awp2 and almost 70 other putative adhesins [14]. Superoxide Dismutase (SOD) enzymes are important virulence factors that play a crucial role in combating oxidative stress during *C. glabrata* infection by mitigating the accumulation of reactive oxygen species (ROS) and prolonging its survival inside phagocytic cells [15]. In *C. glabrata*, proteases play important roles in nutrient stress response, autophagy, azole resistance, and pathogenicity, contributing to the yeast’s survival and clinical challenges [16]. Additionally, *C. glabrata* possesses secreted and membrane-bound virulence factors, including agglutinin-like sequences, heat shock proteins, phospholipases, secreted aspartyl proteinases, lipases, enolases, and phytases, that are hydrolases facilitating fungal invasion, damage, translocation, and immune evasion by interacting with or degrading enterocyte membrane components [17]. Unfortunately, available candidiasis treatment options are limited, and only a few drug classes qualify as antifungal candidates, unlike for bacterial antibiotics. This is due to the fact that both fungi and humans are eukaryotes with high degree of functional orthology and mechanism similarity which makes it hard to develop an anti-fungal drug that only harms the fungus rather than the host [18]. The five main classes of antifungal drugs are: polyenes, azoles, allylamines, flucytosine, and echinocandins [19]. Because of their high bioavailability and water solubility, and low affinity to related human eukaryotic cell membrane plasma proteins, azoles are widely used as the first line of treatment of *Candida* infections in hospitals and healthcare facilities [20]. Azoles target the cytochrome P450 lanosterol 14α-demethylase which converts lanosterol to ergosterol [21]. *ERG11* is the gene that codes for it in yeast. *ERG11* gene mutation induces loss of function and azole resistance by changing the enzyme conformation thus preventing the drug from binding [22]. In some cases, mutations prevent ergosterol formation, allowing the switch to other types of sterols like lanosterol or fecosterol that are not inhibited by drugs [23]. Another mechanism of drug resistance is the overexpression of *ERG11* which leads to higher levels of the target enzyme that overwhelm the inhibitory effects of azoles and subsequently lead to resistance [24]. The overexpression of efflux pumps *CDR1*, *CDR2,* and *MDR1* can also result in resistance as those transporters pump the antifungal drug outside the cell, reducing the intracellular concentration of the drug, and diminishing their ability to inhibit fungal growth [25]. Another mechanism is through mutations in the transcription factor pleiotropic drug-resistance gene *PDR1* which lead to the overexpression of efflux pumps genes [18]. Moreover, heat shock proteins can affect drug resistance [26].

The aim of this study is to determine the cell wall proteomic profile of four *C. glabrata* isolates, with varying degrees of resistance to fluconazole, that have been isolated from a tertiary hospital in Lebanon to identify proteins functioning in virulence and drug resistance. Two of the isolates (8 and 28) have been previously deemed virulent, and two (4 and 29) avirulent [27]. Isolates were grown either in the presence or absence of fluconazole and their respective CWPs extracted and identified via tandem mass spectrometry coupled with a bioinformatics approach. Differentially detected proteins account for the variation in phenotypes. In our lab, we previously carried out such comparative proteomic analyses to characterize some *C. albicans* proteins: Dse1 [28], Pga1 [29], Pir32 [30], Hwp2 [31], and Ddr48 [32]. We used the same approach to identify CWPs present in a *C. albicans* fluconazole-resistant strain from a Lebanese hospital patient upon drug exposure [33].

## Materials and methods

### Isolates utilized in this study

Four C. *glabrata isolates* that were previously recovered from clinical settings and stored in cryobanks at -80°C in 15% glycerol were used for this study. The isolates were obtained from hospitalized patients in tertiary care centers in Lebanon. Ethical approval had been previously obtained from the Lebanese American University IRB board (IRB# LAU.SOM.RH1.26/Apr/2016) [27]. The highly resistant ones designated by isolates 28 and 8 had an MIC of 128 mg/ml, while the less resistant ones designated by isolates 4 and 29 had MIC of 64 mg/ml. Virulence in a disseminated model of murine candidiasis varied from highly avirulent (0/6 deaths for isolates 4 and 29) to virulent for isolate 28 (3/6 deaths), and highly virulent for isolate 8 (5/6 deaths) [27].

Isolates were inoculated in 5 mL potato dextrose broth (Conda Laboratories) and incubated at 30°C for 24 h with shaking at 100 rpm. Isolates were then plated on potato dextrose agar (Conda Laboratories) and incubated at 30°C for 48 hours.

### Growth

Isolates were grown in PDA media for 48 hours 37° C with 100 rpm shaking mode. Cells were cultured either in the presence or absence of fluconazole during this time interval. The concentration of fluconazole added was equivalent to half the MIC for each of the azole susceptible or resistant isolate [33]. Fluconazole was prepared by dissolving 40mg in 2 ml of absolute ethanol as per manufacturer’s recommendation.

### Cell wall isolation and protein extraction

At least 3 independent cell wall extractions from each strain and for each condition were performed. Cell wall was extracted as follows. Cells were first centrifuged at 4,000 rpm for 5 min, then re-suspended in 5 mL Tris (5 mM, pH = 7.8). They were divided into Eppendorf tubes with 1 ml each and spun 2-3 min at 12000 rpm cold, then resuspended in 400 µ L Tris protease inhibitor cocktail (6 µ L, abcam ab65621), along with cold glass beads. 30 cycles of vortexing was done to ensure breakage with samples turning orange, reflecting a reaction between the acidic cytosol and the protease inhibitor. Multiple Eppendorf tubes from one culture were poured in one large tube and the efficiency of the breakage was monitored under the microscope. Supernatants were transferred to pre-weighed tubes. Beads were washed multiple times with 3-4 mL of cold 1 mM NaCl to collect as much cell wall material and proteins. Centrifugation was applied for 5 min at 3000 rpm cold and the supernatant containing the intracellular material was poured off. Pellets were re-suspended in cold NaCl (40 mL, 1 M) and were washed 3-4 times.

The SDS extraction buffer (50 mM Tris, 2% SDS, 100 mM Na-EDTA, 150 mM NaCl, pH 7.8) with β-ME (8 μL per 1 mL SDS extraction buffer) was added (0.5 mL buffer per 100 mg wet weight walls) to the pellets. Samples were boiled at 100°C and then cooled at room temperature. Samples were spun for 5 min 3000 rpm cold and the supernatants containing SDS- extractable protein were collected for further analysis. SDS extraction buffer and β-ME was added again to re-suspend pellet as before. Samples were boiled, cooled, and spun for 5 min at 3000 rpm and suspended in water. Wash steps with water were performed to remove excess SDS. The final pellet was frozen in liquid N2 and freeze-dried. Lyophilized cell walls were stored at − 20°C until use.

### Extraction of alkali labile CWPs

Cell wall pellets were incubated overnight with NaOH (30 mM) at 4°C. They were then neutralized with aqueous acetic acid (30 mM) [34]. Each 4mg of cell wall pellet require 91 μL of NaOH and 109 μL of acetic acid to neutralize them. Samples were spun and supernatants were collected as the alkali labile CWP fraction to be subjected to tryptic digestion.

### Glucanase treatment of cell wall pellets

To maximize the extraction of CWPs, 10^8^ cell equivalents were treated with 1 mg of B-(1-3)-D-Glucanase in sodium acetate buffer (1 mL, 150 mM, pH 5) at 37°C overnight [35]. Cell numbers were estimated using spectrophotometric analysis at a wavelength of 600 nm. The collected supernatants were subjected to tryptic digestion.

### Tryptic digestion

The cell wall extracts were incubated at 55 °C for 1 hr. in a reducing buffer (10 mM DTT, 100 mL NH_4_HCO_3_ Samples were cooled to room temperature and spun. Pellets were resuspended in an alkylating buffer (65 mM iodoacetamide, 100mM NH_4_HCO_3_) and incubated in the dark for 45 min at room temperature. Quenching solution (55 mM DTT, 100 mM NH_4_HCO_3_) was added to the samples subsequently and incubated for 5 min at room temperature. This step was followed by washing the samples 5 times with ammonium bicarbonate buffer (50 mM). Pellets were re-suspended in solution containing ammonium bicarbonate (50 mM) and trypsin (1 µg/ µ L) and incubated for 16 h at 37 °C. They were then spun, and the supernatants were collected and prepared for Zip Tipping by adding TFA 0.1% volume-to-volume ratio.

### Peptide concentration

ZipTip C18 clean up tips (Millipore^®^ Ziptips, Sigma-Aldrich, 0.6 μL C18 resin, volume 10 μl) were wetted in acetonitrile solution and then equilibrated with 0.1% TFA HPLC water solution. The membrane was then washed in a 0.1% TFA HPLC water solution. By using 10 μL of elution buffer (0.1% TFA volume to volume ratio in HPLC water/acetonitrile one-to-one ratio), sample elution was performed.

### Tandem mass spectrometry

Peptides were spotted on a stainless-steel target plate (Opti-TOF TM 384 Well Insert, 128 ×  81 mm RevA, Applied Biosystems), overlaid with α-cyano-4-hydroxy-cinnamic acid matrix solution (10 mg CHCA matrix in 50% acetonitrile with 0.1% TFA), and left to air-dry. MALDI-TOF-TOF MS spectra were obtained using the 4800 MALDI-TOF-TOF analyzer that operates using the 4000 Series Explorer software (version 3.7). TOF/TOF Calibration Mixture (Mass Standards Kit for Calibration of AB SCIEX TOF/TOF Instruments) was used to externally calibrate the machine. For acquisition, MS reflector positive mode at a laser intensity of 2500 was used. The selected mass range was 499-2500 Da with a focus mass of 1500 Da. Reflector positive default was used as a processing method. The minimum signal-to-noise ratio was set at 5. The obtained mass lists were manually searched for recognized contaminant mass peaks (matrix, keratin, and trypsin autolysis). Accordingly, an exclusion list was formed and incorporated in all executed MS/MS data acquisitions. Regarding the interpretation method, MS/MS 1 kV positive was applied as an MS/MS acquisition method at a laser intensity of 3500 and a precursor mass of 1570.677 Da while turning on the metastable suppressor and CID, and MS/MS positive default was applied as an MS/MS processing method with a signal-to-noise limit of 5 for monoisotopic peaks.

### Protein identification

MS/MS Ion Search was performed using the contaminants and CRAP databases to remove any additional contaminants whose peaks were not included within the exclusion list. MASCOT search engine (version 2.5.1) was used in both MASCOT Server software and ProteinPilot software for protein identification. A custom *C. glabrata* database was created and used to carry out the MS/MS Ion Search. The database contained protein sequences of all curated proteins in the Swissprot database (retrieved on January 14, 2024), which is a total of 890 sequences to be searched. The peptide and fragment tolerance values were specified at ± 2 Da. We had to choose this lenient tolerance because the 4800 MALDI-TOF-TOF analyzer was displaying a low resolution per mass peak, a limitation of our machine. Carbamidomethyl C was assigned as a fixed modification and Oxidation at M was chosen as a variable modification. Up to two missed cleavages were allowed for trypsin. Peptide charge of + 1 was selected and the instrument type was allocated as MALDI-TOF-TOF. In the obtained summary reports, proteins were considered successful hits if their score exceeded the threshold calculated by the engine and if their peptide sequence was at least 6 amino acids long. Similarly, this peptide length was set as a criterion for identification in a recent study conducted by Chew et al. for the proteomic profiling of acetate-grown *C. glabrata* [36]. Peptide sequences identified by MASCOT search engine but not linked to proteins were blasted on Candidagenome.org. The selected target genome and target sequence dataset were “*Candida glabrata* CBS138 Assembly” and “Proteins – translation of coding sequence (PROTEIN)” respectively. The BLASTP program was selected, and no gapped alignments were allowed. For each blasted sequence, the protein hit with highest score, 100% identity, and e-value <  0.05 was chosen. The cutoff E-value was set at < 0.05 for MASCOT searches as well as BLAST searches. All of these factors combined increased confidence in our results especially that when a peptide was short (6-7 amino acids long), the respective protein was identified using more than one peptide and peptides were identified using more than one source (MASCOT, ProteinPilot, and/or BLASTP). When one peptide was used for identification, the peptide was at least 8 amino acids in length.

## Results

### Proteins involved in virulence

To find out which proteins play a role in the virulence of *C. glabrata*, cell wall proteome of isolates 8 and 28 that are virulent and isolates 4 and 29 that are avirulent were compared to each other both in the presence and absence of fluconazole; differentially-detected proteins in the virulent strains were reported.

### CWPs exclusive to C. *glabrata* virulent strains when exposed to fluconazole

Our results show that, after exposure to fluconazole, 25 proteins were identified in the virulent strains but not in the avirulent strains (Table 1). We identified several key proteins implicated in fluconazole resistance and virulence modulation. Notable among these were proteins associated with autophagy process. Among the major proteins identified were Cdr1, Alg2, Mcd4 and Hhf. More details regarding those proteins and their source of detection are available in S1 Table.

**Table 1:** Proteins identified exclusively in *C. glabrata* virulent strains (8 and 28) as compared to the avirulent strains (4 and 29) when grown in the presence of fluconazole.

Protein Accession	Gene Name	Protein Description
P60009	ACT1	Actin
Q6FJJ9	ALG2	Alpha-1,3/1,6-mannosyltransferase
Q6FPI6	ATG15	putative lipase ATG15 (EC 3.1.1.3) (Autophagy-related protein 15)
Q6FP05	ATG2	Autophagy-related protein 2
Q6FRR4	ATG32	Autophagy-related protein 32
Q6FJZ6	ATG5	Autophagy protein 5
Q6FTM0	CAGL0G01430g	Leucine aminopeptidase 2
Q6FK23	CDR1	Pleiotropic ABC efflux transporter of multiple drugs CDR1
Q6FSM8	FYV8	Protein FYV8
Q8NIG3	HHF1	Histone H4
Q6FLD4	ISN1	IMP-specific 5’-nucleotidase 1
Q6FS62	LCL3	Probable endonuclease LCL3
Q6FJ81	MCD4	GPI ethanolamine phosphate transferase 1
Q6FJA3	PDC1	Pyruvate decarboxylase
Q6FKK0	RIM20	pH-response regulator protein
Q6FTK4	RPL11	Large ribosomal subunit protein uL5
Q6FNN1	RPL28	Ribosomal protein L28 of the large subunit
Q6FR56	RPS16	Small ribosomal subunit protein uS9
Q6FY30	SBE2	Protein SBE2
Q6FKC2	SGF11	SAGA-associated factor 11
Q6FLL2	SKG1	Ortholog of *S. cerevisiae*: AIM20
Q00372	SNF1	Carbon catabolite-derepressing protein kinase
Q6FVM9	SRB8	Mediator of RNA polymerase II transcription subunit 12
Q6FNJ6	TDA7	Topoisomerase I damage affected protein 7
Q6FWW3	ZRG8	Zinc-regulated protein 8

### CWPs exclusive to *C. glabrata* virulent strains when grown in the absence of fluconazole

Comparison of the cell wall proteomic profiles of fluconazole-unexposed *C. glabrata* virulent and avirulent strains showed that only four proteins were solely identified in the virulent strains (Table 2). This is significantly lower than the number of proteins differentially detected upon fluconazole exposure. More details regarding those proteins and their source of detection are available in S2 Table.

**Table 2:** Proteins identified exclusively in *C. glabrata* virulent strains (8 and 28) as compared to the avirulent strains (4 and 29) when grown in the absence of fluconazole.

Protein Accession	Gene Name	Protein Description
Q6FMR7	CAGL0K05775g	Succinate dehydrogenase assembly factor 3, mitochondrial
Q6FNU6	CPR6	Peptidyl-prolyl cis-trans isomerase D
Q6FYA7	EFT1	Elongation factor 2
Q6FSN5	OXR1	Oxidation resistance protein 1

### Proteins involved in drug resistance

To highlight the proteins involved in fluconazole resistance of *C. glabrata* isolates, each of the 4 isolates was grown in the presence and absence of fluconazole. The cell wall proteomic profiles of each isolate under both growth conditions were compared focusing on the differentially detected proteins in the fluconazole-exposed isolates.

### Differentially identified CWPs in fluconazole-exposed *C. glabrata* strain 4

Ten proteins were differentially identified in strain 4 under fluconazole growth condition and those proteins are responsible for the observed resistance in this strain (Table 3). More details regarding those proteins and their source of detection are available in S3 Table.

**Table 3:** Number of proteins exclusively found in strain 4 under exposure to fluconazole.

Protein Accession	Gene Name	Protein Description
Q6FRZ8	AP1	bZip transcription factor
Q6FMU9	CAGL0K05027g	Adenylosuccinate synthetase
Q6FTW6	ENO1	Enolase 1
Q6FK71	FPR4	FK506-binding protein 4
P61833	HHT1	Histone H3
O74208	PDH1	Pleiotropic ABC efflux transporter of multiple drugs
Q6FNY7	POL2	DNA polymerase epsilon catalytic subunit A
Q6FIS9	PYK1	Pyruvate kinase 1
Q6FN94	RPN4	C2H2-type transcription factor
O93796	TEF3	Elongation factor 3

### Differentially identified CWPs in fluconazole-exposed *C. glabrata* strain 8

A total of 34 proteins were differentially identified in strain 8 upon fluconazole exposure (Table 4). Many of the proteins relate to autophagy, and lipase enzymes that are known to contribute to virulence. More details regarding those proteins and their source of detection are available in S4 Table.

**Table 4:** Number of proteins exclusively found in strain 8 under exposure to fluconazole.

Protein Accession	Gene Name	Protein Description
Q6FVR8	RIM8	pH-response regulator protein palF/RIM8
Q6FK23	CDR1	Pleiotropic ABC efflux transporter of multiple drugs CDR1
Q6FPJ5	FMP29	Altered inheritance of mitochondria protein 9, mitochondrial
P60009	ACT1	Actin
Q6FJJ9	ALG2	Alpha-1,3/1,6-mannosyltransferase ALG2
Q6FPI6	ATG15	Putative lipase ATG15
Q6FJZ6	ATG5	Autophagy protein 5
Q6FTM0	CAGL0G01430g	Leucine aminopeptidase 2
Q6FJ69	CAGL0M08734g	FAS1 domain-containing protein
Q6FVB6	CHO2	Phosphatidylethanolamine N-methyltransferase
Q6FY24	CLN1	Biogenesis of lysosome-related organelles complex 1 subunit
Q6FWU4	DSE3	Protein DSE3
Q6FKB4	EIS1	Eisosome protein 1
Q6FM00	EXO84	Exocyst complex component EXO84
Q6FK71	FPR4	FK506-binding protein 4
Q6FSM8	FYV8	Protein FYV8
Q8NIG3	HHF1	Histone H4
Q6FJ81	MCD4	GPI ethanolamine phosphate transferase 1
Q6FYA6	MUS81	Crossover junction endonuclease MUS81
Q6FJA3	PDC1	Pyruvate decarboxylase
Q6FIS9	PYK1	Pyruvate kinase 1
Q6FKK0	RIM20	pH-response regulator protein
Q6FTK4	RPL11	Large ribosomal subunit protein uL5
Q6FR56	RPS16	Small ribosomal subunit protein uS9
Q6FY30	SBE2	Protein SBE2
Q6FRV6	SEC16	COPII coat assembly protein SEC16
Q6FLL2	SKG1	Ortholog of *S. cerevisiae*: AIM20
Q00372	SNF1	Carbon catabolite-derepressing protein kinase
Q6FVM9	SRB8	Mediator of RNA polymerase II transcription subunit 12
Q6FNJ6	TDA7	Topoisomerase I damage affected protein 7
Q6FSM4	TDH3	Glyceraldehyde-3-phosphate dehydrogenase 2
O93796	TEF3	Elongation factor 3
Q6FRH8	VPS15	non-specific serine/threonine protein kinase
Q6FWW3	ZRG8	Zinc-regulated protein 8

### Differentially identified CWPs in fluconazole-exposed *C. glabrata* strain 28

In total, 19 proteins were differentially detected in strain 28 in the presence of fluconazole. Those proteins contribute to the observed phenotypes of increased virulence and resistance to both fluconazole and echinocandins (Table 5). More details regarding those proteins and their source of detection are available in S5 Table.

**Table 5:** Number of proteins exclusively found in strain 28 under exposure to fluconazole.

Protein Accession	Gene Name	Protein Description
Q6FJA3	PDC1	Pyruvate decarboxylase
Q6FRH2	ATG11	Autophagy-related protein 11
Q6FP05	ATG2	Autophagy-related protein 2
Q6FRR4	ATG32	Autophagy-related protein 32
Q6FVB6	CAGL0E03201g	Phosphatidylethanolamine N-methyltransferase
Q6FTK4	CAGL0G01826g	Large ribosomal subunit protein uL5
Q6FS62	CAGL0H03201g	Probable endonuclease LCL3
Q6FJZ6	CAGL0M02343g	Autophagy protein 5
Q6FJ38	CAGL0M09471g	Autophagy protein 16
Q6FQY4	ENO2	Enolase 2
Q6FSM8	FYV8	Protein FYV8
Q8NIG3	HHF1	Histone H4
Q6FK84	IML1	Vacuolar membrane-associated protein
Q6FLD4	ISN1	IMP-specific 5’-nucleotidase 1
Q6FJ81	MCD4	GPI ethanolamine phosphate transferase 1
Q6FKK0	RIM20	pH-response regulator protein
Q6FR56	RPS16	Small ribosomal subunit protein uS9
Q6FKC2	SGF11	SAGA-associated factor 11
Q6FSR7	VPS34	Phosphatidylinositol 3-kinase

### Differentially identified CWPs in fluconazole-exposed *C. glabrata* strain 29

In addition, twenty-four proteins were identified in strain 29, notable proteins are Eno2, Atg14, Atg16, and Pup1 (Table 6). More details regarding those proteins and their source of detection are available in S6 Table.

**Table 6:** Number of proteins exclusively found in strain 29 under exposure to fluconazole.

Protein Accession	Gene Name	Protein Description
Q6FX35	ATG14	Autophagy-related protein 14
Q6FJ38	ATG16	Autophagy protein 16
Q6FRM0	CAGL0H0 7513g	Very-long-chain 3-oxoacyl-CoA reductase
Q6FVC7	CGR1	rRNA-processing protein
Q6FVB6	CHO2	Phosphatidylethanolamine N-methyltransferase
Q6FYA7	EFT2	Elongation factor 2
Q6FKB4	EIS1	Eisosome protein 1
Q6FQY4	ENO2	Enolase 2
Q6FJW2	EXO70	Exocyst complex protein
Q6FK71	FPR4	FK506-binding protein 4
Q6FJX6	HHOA	Histone H1
Q6FK84	IML1	Vacuolar membrane-associated protein
Q6FJW0	IRS4	Increased rDNA silencing protein 4
Q6FU50	LHS1	Heat shock protein 70 homolog LHS1
Q6FIK6	LIP1	Ceramide synthase subunit
Q6FKJ1	NTE1	Lysophospholipase NTE1
Q6FKY1	PGK1	Phosphoglycerate kinase
Q6FUI5	PPM1	Leucine carboxyl methyltransferase 1
Q6FIN8	PUP1	PDR1 up-regulated protein 1
Q6FV12	PYK2	Pyruvate kinase 2
Q6FSN6	RPL7	Large ribosomal subunit protein uL30
Q6FRV6	SEC16	COPII coat assembly protein SEC16
Q6FRH8	VPS15	non-specific serine/threonine protein kinase
Q6FP39	VPS30	Vacuolar protein sorting-associated protein 30

## Discussion

*C. glabrata* is an emerging fungal pathogen that has not been extensively studied as much as its more clinically significant cousin, *C. albicans* with most studies focusing on the genomics of the organism and only few focusing on proteomic profiling. This study aimed to identify CWPs that are responsible for virulence and fluconazole resistance in *C. glabrata* isolates from Lebanese hospital. Strains 8 and 28 were previously shown to be highly virulent, whereas strains 4 and 29 were found to be avirulent. Moreover, strains 8 and 28 were previously found to be more resistant to fluconazole than strains 4 and 29 [27]. It is important to note that the lack of detection of a protein in a sample does not necessarily imply that this protein is strictly absent; it could still be present but below detection threshold. Even so, the exclusive detection of a protein in a sample is still significant suggesting that one strain likely has higher levels of the identified protein compared to the other. Such variations are responsible for the differences in the observed phenotypes across the studied strains. To increase confidence in our results, proteins were identified via multiple sources (MASCOT, ProteinPilot, and/or BLASTP) using more than one peptide when peptide length was 6-7 amino acids, or using one peptide when peptide length was at least 8 amino acids. Also, while the majority of the detected proteins are CWPs, some are cytoplasmic or nuclear. Some of these mitochondrial or ribosomal subunit proteins could be a result of contamination from the accidental rupture of the protoplast during extraction, yet they could possibly be atypical proteins with a novel role in the cell wall. When exposed to harsh growth conditions, *C. glabrata* dynamically adapts by changing the cell wall structure and proteome with “moonlighting proteins” being exposed on the surface to facilitate adhesion and pathogenicity [37]. They could also be components of extracellular vesicles. *Candida* vesicles carry key virulence factors like Als family adhesins, secreted aspartic proteinases, moonlighting proteins, and RNA molecules that might influence gene expression in host cells [38]. Extracellular vesicles and their components play a crucial role in many processes such as adhesion to different surfaces, infection progression, biofilm formation, formation of a complex community through facilitating communication within cells of the same species [39]. They can also interact with other microorganisms in the same niche which could lead to multispecies infections [40].

In the presence of fluconazole, proteins detected solely in the virulent strains have implications for both virulence and drug resistance. Hhf1 is interesting since the intracellular proliferation of *C. glabrata* is dependent upon its ability to remodel its chromatin, reprogram its carbon metabolism and induce autophagy [41]. The modulation of chromatin structure by Hhf1, which is detected in the fungal biofilm matrix and the nucleosome, affects how genes are expressed, influencing the transcriptional activity of genes that encode for adhesins, secreted hydrolytic enzymes, and stress response factors [4]. These processes facilitate *C. glabrata*’s ability to form biofilms on medical devices and tissues, contributing to chronic infections and resistance to treatments. Sgf11 modulates histone deubiquitination, biofilm formation, oxidative stress response, drug tolerance, and cell wall integrity [42]. Thus, Sgf11 contributes to *C. glabrata*’s persistence in the host and response to treatment. Alg2 is integral to the biosynthesis of the cell wall, a structure vital for the yeast’s survival and interaction with the host, as this protein catalyzes the mannosylation step necessary for the biosynthesis of N-linked glycans [43,44]. Mcd4 is an ethanolamine phosphate transferase involved in GPI-anchor biosynthesis. This protein is important since many of the CWPs in *C. glabrata* are GPI-anchored proteins allowing the yeast to interact with the host and environment [13]. As such, Mcd4 is a potential target for therapeutic strategies. Moreover, Sbe2 and Skg1 were solely detected in the virulent strains and they play role in cell wall integrity. Cell wall integrity and its ability to undergo modifications are essential for *C. glabrata* to adhere to host tissues, form biofilms, and evade the host immune system [45].

Furthermore, metabolic adaptability of *C. glabrata* is a key factor in its ability to thrive as a pathogen and for its invasive growth. *C. glabrata* possesses adhesins that promote tissue colonization and invasion and we were able to detect Tda7, a putative adhesin-like protein [46]. Adhesins are crucial for mediating the initial interaction between *C. glabrata* and the host surface that can subsequently lead to a persistent infection especially in immunocompromised patients [47]. In a recent study, we reported that azole-resistant *C. glabrata* strains possess mutations in subtelomeric silencer Sir3 that de-represses adhesins leading to increased adhesion and more stable biofilm formation in those strains when compared to the azole-susceptible strains [48]. The enzyme pyruvate decarboxylase (Pdc1) plays a pivotal role in fermentative metabolism, energy production, and interaction with the host [49]. Even more, Rogers et al. [50] found that Pdc1 levels were increased in azole resistant strain hinting for a role in azole resistance. Likewise, we found this protein exclusively in the virulent strains that are as well more resistant to fluconazole, making it a potential target for therapeutic intervention. Snf1 plays a major role in glucose metabolism and the utilization of alternative carbon sources under starvation [51]. In addition to that, the proteins Atg15, Atg2, Atg32, and Atg5 are lipases and are part of the autophagy pathway, a process that the cell uses to degrade and recycle cellular components [52]. Autophagy can help the pathogen survive in hostile environments, affect cell wall composition, and regulate factors that directly contribute to virulence [53]. ATG proteins have been implicated in resistance to oxidative stress [54]. Unsurprisingly, those ATG proteins were exclusively detected in the virulent, more fluconazole-resistant strains. Similarly, Amador-García et al. revealed an increase in the abundance of proteins functioning in oxidative stress response, protein folding, and proteasome-dependent catabolism in *C. albicans* when exposed to oxidative stress [55]. Hydrolases, that play a crucial role in tissue adhesion and penetration and are responsible for host invasion [56], were also exclusively detected in those strains; mainly CAGL0G01430g, Isn1, and Lcl3. These hydrolases facilitate tissue invasion by degrading host extracellular matrix components [17], allowing for invasive infections that are challenging to treat clinically. An important protein exclusively detected in the virulent strains is Rim20 whose regulatory functions enable the yeast to transition from commensalism to pathogenicity, adjusting gene expression to optimize survival in acidic or neutral pH conditions encountered within the host [57].

Moreover, Rpl11 and Rpl16 are both ribosomal proteins that we assume to be moonlighting proteins with novel roles in the cell wall and contributing in virulence. Xu et al. [58] reported those proteins in samples from fungal biofilm matrix as well. Their localization in the fungal biofilm matrix suggests potential involvement in biofilm formation and persistence [44]. Therefore, Rpl11 and Rpl16 are clinically important in persistent infections especially that biofilms are the foundation of chronic infections and problematic to eradicate [59]. Gong et al. identified few moonlighting proteins in *C. parapsilosis* cell wall proteome and hypothesized that they could be potential biomarkers for the development of vaccines against *C. parapsilosis* [60]. In the same context, we assume that those moonlighting proteins that we detected can be used as a vaccine against *C. glabrata*. Moreover, a recent study identified that the Rpl class of proteins play an important role in aerobic respiration in *C. glabrata* and at the same time act an antigens in secretory proteins. It also suggested that those Rpl antigenic proteins can be used as *C. glabrata* biomarkers and candidates for therapeutics [61]. Other proteins with cellular location being the fungal biofilm matrix detected in our virulent strains are Act1 that plays a role in cell motility, and Zrg8 that contributes to polarized growth and fungal cell wall organization [58]. All those proteins collectively contributed to the virulence of the studied strains 8 and 28 and their lack of detection in the other 2 strains, 4 and 29, explain their avirulent phenotype. When compared in the absence of fluconazole, only 4 proteins were exclusively detected in the virulent strains: CAGL0K05775g, Cpr6, Eft1, and Oxr1. Those proteins are not well studied for their role in the cell wall; suggesting a novel function in *C. glabrata* virulence and cell wall localization. Also, given the discrepancy in the number of detected proteins in the absence and presence of fluconazole, this gives additional proof that the cell wall dynamically changes its proteome when exposed to the drug to ensure survival.

For that reason, we aimed to additionally compare the cell wall proteome of each strain in the presence and absence of fluconazole to identify strain specific proteins involved in response to drug exposure. The exclusive detection of Cdr1 (strain 8) and Cdr2 (strains 4 and 29) upon fluconazole exposure was expected since the overexpression of efflux pumps is a principal mechanism of drug resistance through which *C. glabrata* can excrete fluconazole and other antifungal drugs, thereby reducing their intracellular concentrations and making them less effective [62]. Efflux pump-mediated drug resistance is a significant clinical problem, adding more challenges to candidiasis treatment and management. Studies are exploring the potential of using fungal pump inhibitors as an azole-enhancing combination therapy [63]. Rpn4, detected in strain 4 under fluconazole expression, controls azole resistance through mediating ergosterol and plasma membrane permeability by regulating proteasome and ergosterol biosynthesis genes including *ERG1*, *ERG2*, *ERG3*, and *ERG11* [64,65]. Pais et al. reported that Rpn4 is a promising target to combat azole resistance due to its direct control over *ERG11* expression and its effect on cell permeability and fluconazole accumulation [64]. Most significantly, Fpr4 was exclusively detected in 3 strains (4, 8, and 29) upon fluconazole exposure as opposed to their unexposed counterparts. Fpr4, a histone chaperone, leads to proline isomerization that influences H3 methylation which in turn regulates multidrug resistance gene expression; thereby, governing azole antifungal resistance [66]. This detected histone chaperone is clinically important. It was previously suggested that allocating agents that target fungal-specific histone modifying enzymes potentially allows the development of new classes of antifungal drugs or enhances the efficacy of current antifungals [67]. Ap1 (strain 4) is involved in multidrug resistance and it is required for the activation of multidrug transporter Flr1 [68].

Pup1 (strain 29) plays a role in increased fluconazole resistance and its gene is upregulated by *CDR1* [69]. Rim8 (strain 8) and Rim20 (strain 8 and 28) are integral components of the pH-responsive signaling pathway, the Rim101 pathway, in *C. glabrata*, and play crucial roles in sensing and responding to environmental stressors like fluconazole exposure, which perturbs pH balance [70]. Rim101 pathway regulates the expression of genes involved in cell wall, adhesion, iron metabolism, and biofilm formation. Phenotypic and transcriptional analyses have noticed the role of multiple components of the Rim pathway in azole tolerance and identified that Hsp90 is a downstream effector of Rim signaling, likely contributing to Rim-mediated effects on antifungal tolerance [57]. A promising antifungal strategy could be to target the Rim pathway along with current antifungal drugs to specifically target Hsp90 [71].

Atg11, Atg16, Atg2, Atg32, and Atg5 detected in strain 28 upon drug exposure mediate important processes such as cytoplasm to vacuole transport, mitophagy, and autophagic remodeling, which are fundamental for stress rescue [72]. In strain 29, Atg14, Vps15 (also detected in strain 8), and Vps30 proteins were detected upon drug exposure. Those proteins are involved in regulating autophagosome structure and have an essential role in intracellular survival of *C. glabrata* [73,74]. By maintaining cellular homeostasis and ensuring the effective removal of damaged organelles and cell debris, they contribute to the adaptation of *C. glabrata* and its resistance to antifungal agents [72,74]. In addition, it has been reported that the regulation of autophagy and other pathways related to starvation processes may have an indirect impact on drug efflux pumps and other resistance mechanisms [75]. Detected proteins related to autophagy and/or survival upon starvation following fluconazole exposure include: Sec16 (strains 8 and 29), Ppm1 (strain 29), Iml1 (strains 28 and 29), Snf1 (strain 8), and Irs4 (strain 29). Furthermore, Pyk1 (strain 4 and 8) and Tdh3 (strain 8) have a role in glycolysis, the primary metabolic pathway for energy production, which is essential for powering drug efflux pump activity and maintaining cell membrane integrity [76]. Studies have shown increased expression of Tdh3 in azole-resistant organisms, with high abundance in biofilms [77]. Similarly, Buakaew et al. revealed an altered response in *C. albicans* proteomic profile upon β-citronellol treatment with significant effect on CWPs, cellular stress response enzymes, and ATP synthesis-associated proteins [78].

Upon fluconazole exposure, some proteins (Fyv8, Hhf1, Mcd4, Pdc1, and Rps16) were only detected in the virulent strains 8 and 28 but not in the avirulent strains 4 and 29. This hints at the important roles these proteins perform in both drug resistance, and virulence. Their roles in virulence are discussed above and their role in fluconazole resistance is yet to be elucidated.

To maximize the pool of detected proteins, we used ProteinPilot server in addition to Mascot and BLAST. ProteinPilot delivers a summarized report with minimal information regarding the protein hits. The exported report does not include peptide sequences and protein coverage though. Furthermore, *C. glabrata* is not a well-studied fungus with a limited number of curated proteins in the Swissprot database and limited published research on its cell wall proteome, virulence and resistance factors. *C. glabrata* has around 6000 genes, hence no less than 6000 proteins. However only around 900 are found in the Swissprot database. In our study, we subjected the cells to planktonic growth conditions rather than opting for biofilm-inducing growth conditions which could have potentially led to a larger pool of extracted CWPs. However, since we were studying the variation in cell wall proteomic profile upon fluconazole exposure, we did not want to impose further stress on the fungal cells that could have interfered with the expressions. In future studies, biofilm-inducing conditions should be added as an additional factor to evaluate differences in proteomic profiles. Despite all these limitations, our pilot study was able to identify many important proteins for virulence like autophagy-related proteins, hydrolases, other proteins related to stress response, membrane signaling and cell wall synthesis. We were also able to identify many proteins related to drug resistance. To our knowledge, this is the first study to explore the cell wall proteomic profile *C. glabrata* isolates with varying degrees of virulence and resistance to fluconazole in the absence and presence of fluconazole drug. Many of the proteins solely detected in the virulent and resistant strains could be potential therapeutic agents for new antifungal therapy or azole-enhancing combination therapy as discussed above. They can as well be used as biomarkers for the development of vaccines against *C. glabrata* infections. This is extremely important given the rise in drug resistance and the burden this holds on immunocompromised individuals in specific. Future work should delve deeper into understanding the interplay between the identified proteins and their roles in conferring azole resistance and modulating virulence in *C. glabrata* strains, and confirm key observed proteomic differences through western blotting, quantitative mass spectrometry, or transcriptional profiling.

## Supporting information

S1 Table Proteins identified exclusively in *C. glabrata* virulent strains (8 and 28) as compared to the avirulent strains (4 and 29) when grown in the presence of fluconazole. Proteins identified using MASCOT are represented with MASCOT score, sequence coverage, missed cleavage, and peptide sequence; proteins identified using BLAST are represented with protein score, peptide sequence, and E-value; proteins identified using ProteinPilot are represented with protein score only.
(XLSX)

S2 Table Proteins identified exclusively in *C. glabrata* virulent strains (8 and 28) as compared to the avirulent strains (4 and 29) when grown in the absence of fluconazole. All differentially expressed proteins were identified using ProteinPilot.
(XLSX)

S3 Table Number of proteins exclusively found in strain 4 under exposure to fluconazole. Proteins identified using MASCOT are represented with MASCOT score, sequence coverage, missed cleavage, and peptide sequence; proteins identified using BLAST are represented with protein score, peptide sequence, and E-value; proteins identified using ProteinPilot are represented with protein score only.
(XLSX)

S4 Table Number of proteins exclusively found in strain 8 under exposure to fluconazole. Proteins identified using MASCOT are represented with MASCOT score, sequence coverage, missed cleavage, and peptide sequence; proteins identified using BLAST are represented with protein score, peptide sequence, and E-value; proteins identified using ProteinPilot are represented with protein score only.
(XLSX)

S5 Table Number of proteins exclusively found in strain 28 under exposure to fluconazole. Proteins identified using MASCOT are represented with MASCOT score, sequence coverage, missed cleavage, and peptide sequence; proteins identified using BLAST are represented with protein score, peptide sequence, and E-value; proteins identified using ProteinPilot are represented with protein score only.
(XLSX)

S6 Table Number of proteins exclusively found in strain 29 under exposure to fluconazole. Proteins identified using MASCOT are represented with MASCOT score, sequence coverage, missed cleavage, and peptide sequence; proteins identified using BLAST are represented with protein score, peptide sequence, and E-value; proteins identified using ProteinPilot are represented with protein score only.
(XLSX)
